# Supplementary figures and images for: Bias in comparisons of mortality among very preterm births: A cohort study
Source: PLoS One. 2021 Jun 30;16(6):e0253931. doi: 10.1371/journal.pone.0253931 (PMC8244917; doi:10.1371/journal.pone.0253931)

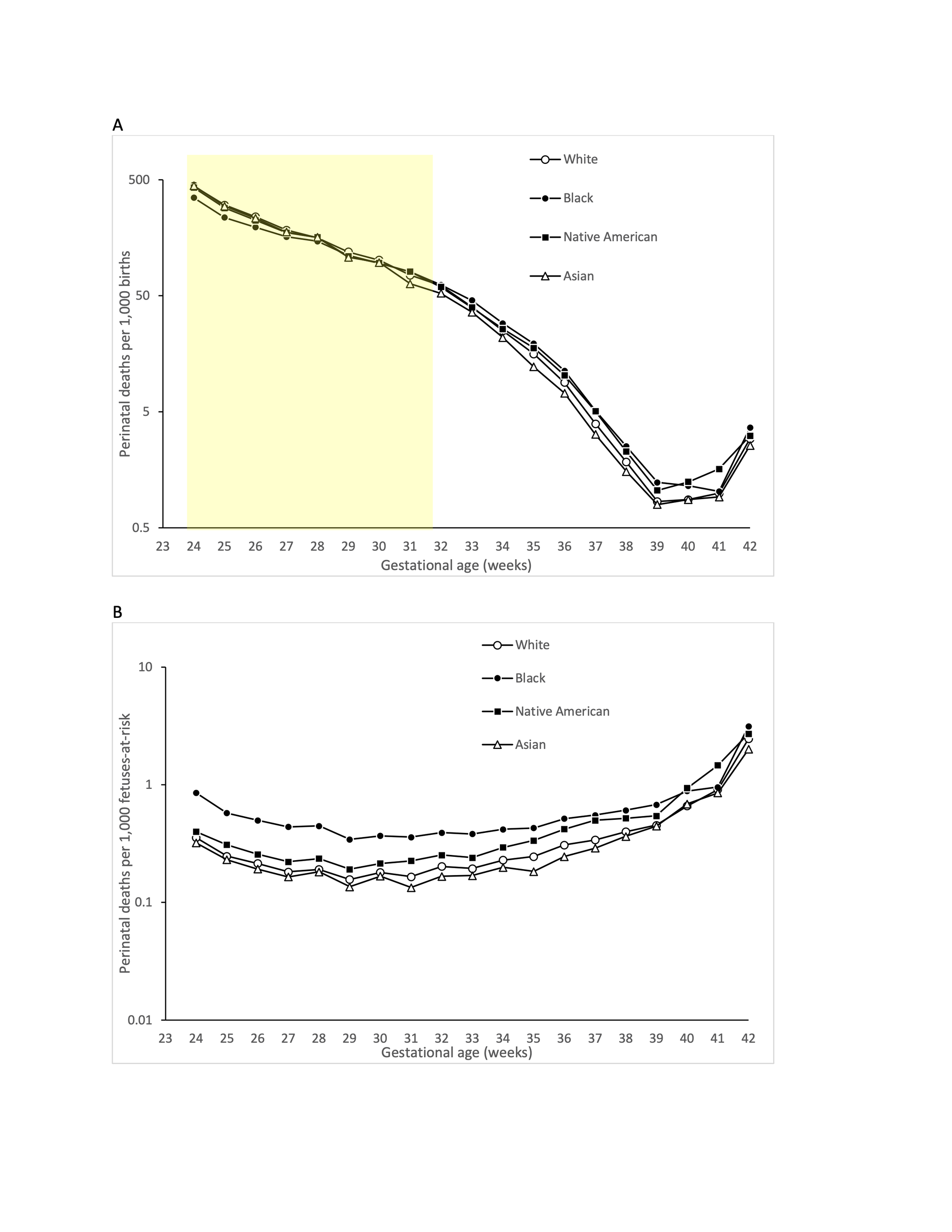

Supplement: S1 Fig — Gestational age-specific perinatal death rates of singletons with no congenital or chromosomal anomalies by maternal race using a births-based denominator (A) or using a fetuses-at-risk denominator (B), United States, 2006–2015. The yellow area highlights the restricted subpopulation at 24–31 weeks’ gestation. (TIF) [file pone.0253931.s001.tif]

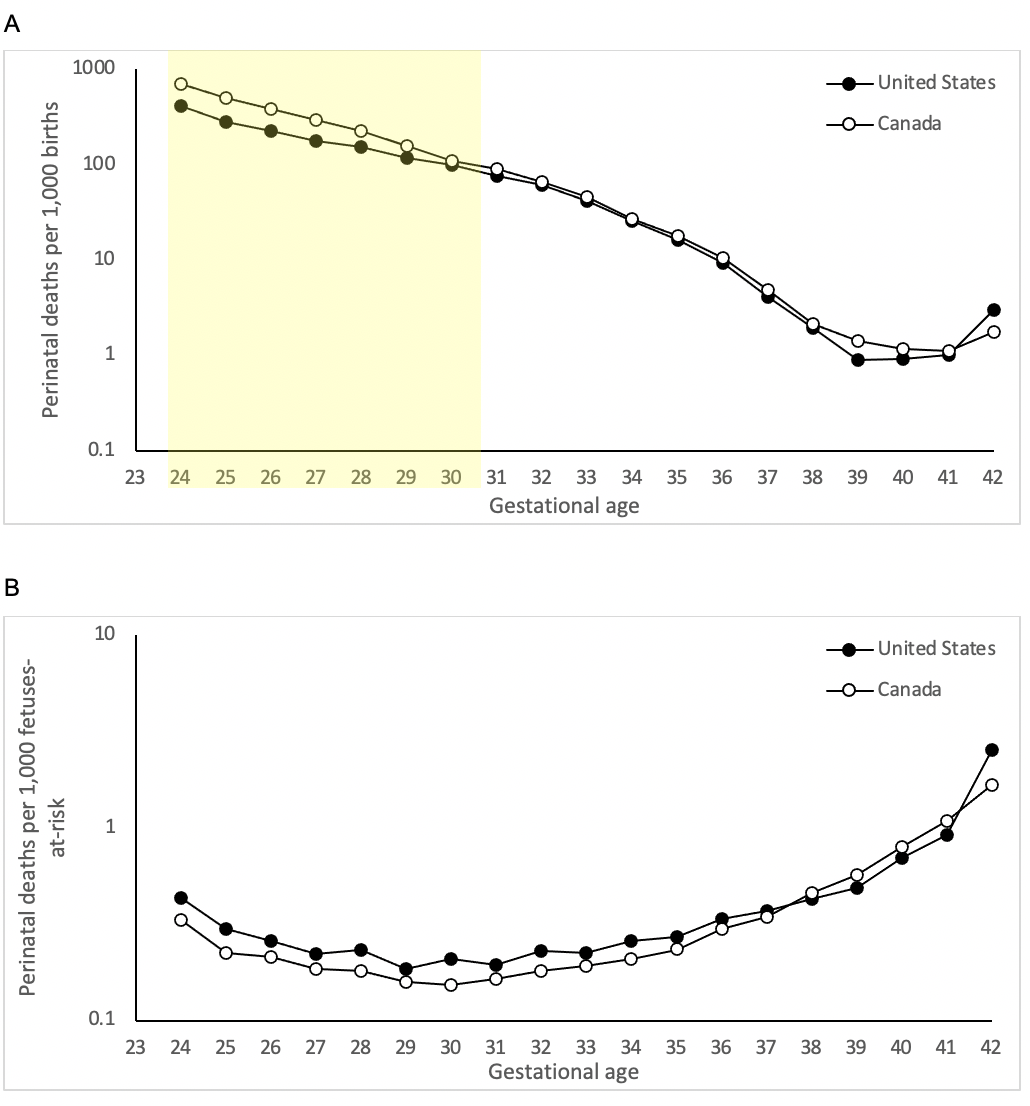

Supplement: S2 Fig — Gestational age-specific perinatal death rates of singletons with no congenital or chromosomal anomalies in Canada or in the United States using a births-based denominator (A) or using a fetuses-at-risk denominator (B), 2006–2015. The yellow area highlights the restricted subpopulation at 24–31 weeks’ gestation. (TIF) [file pone.0253931.s002.tif]

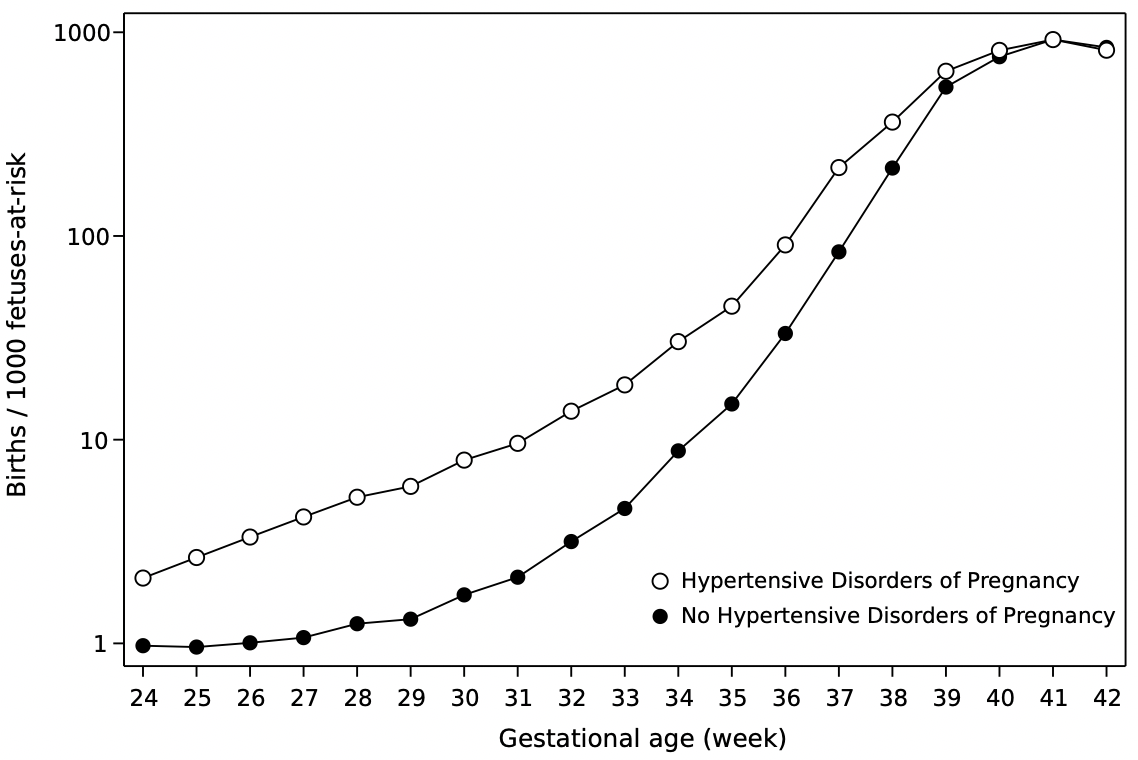

Supplement: S3 Fig — (TIF) [file pone.0253931.s003.tif]

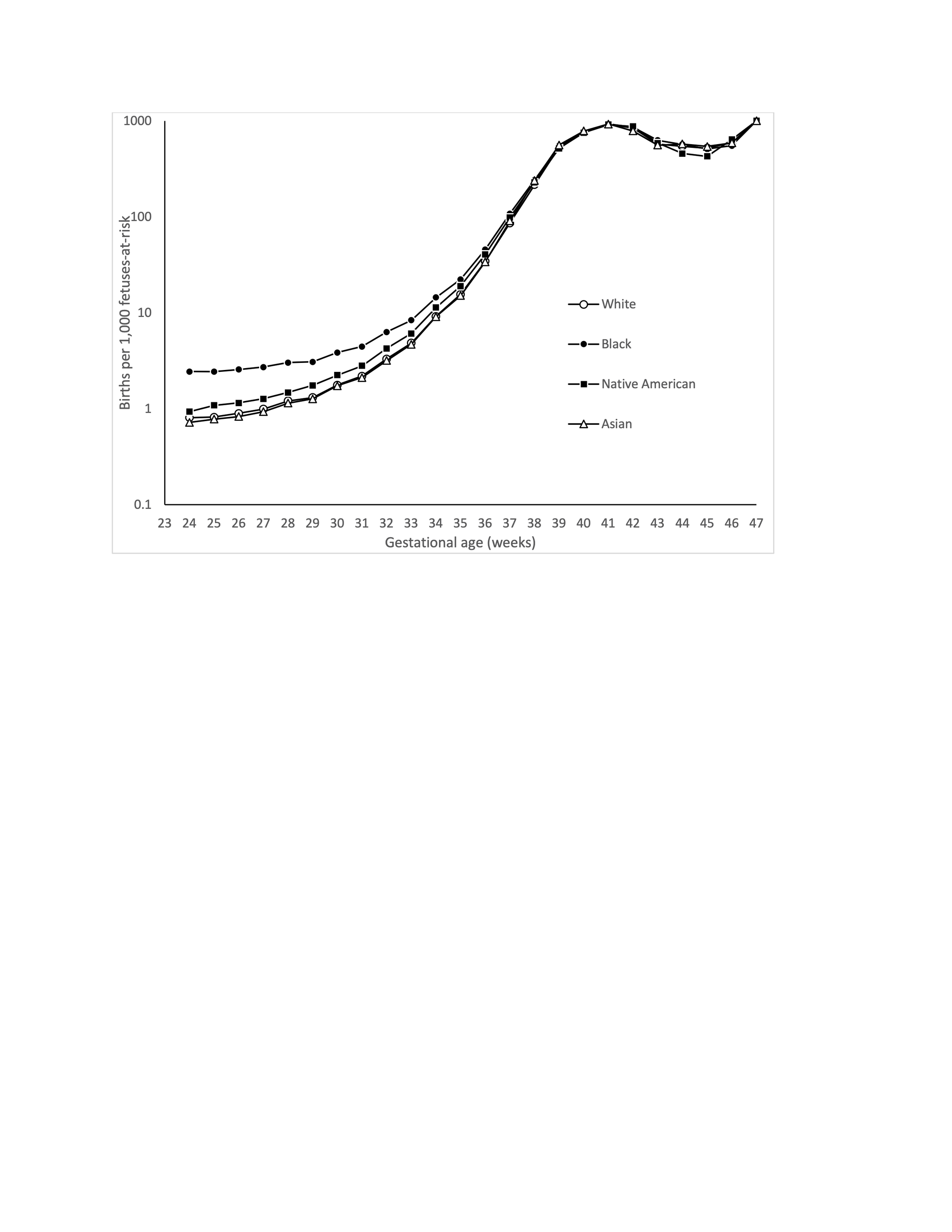

Supplement: S4 Fig — (TIF) [file pone.0253931.s004.tif]

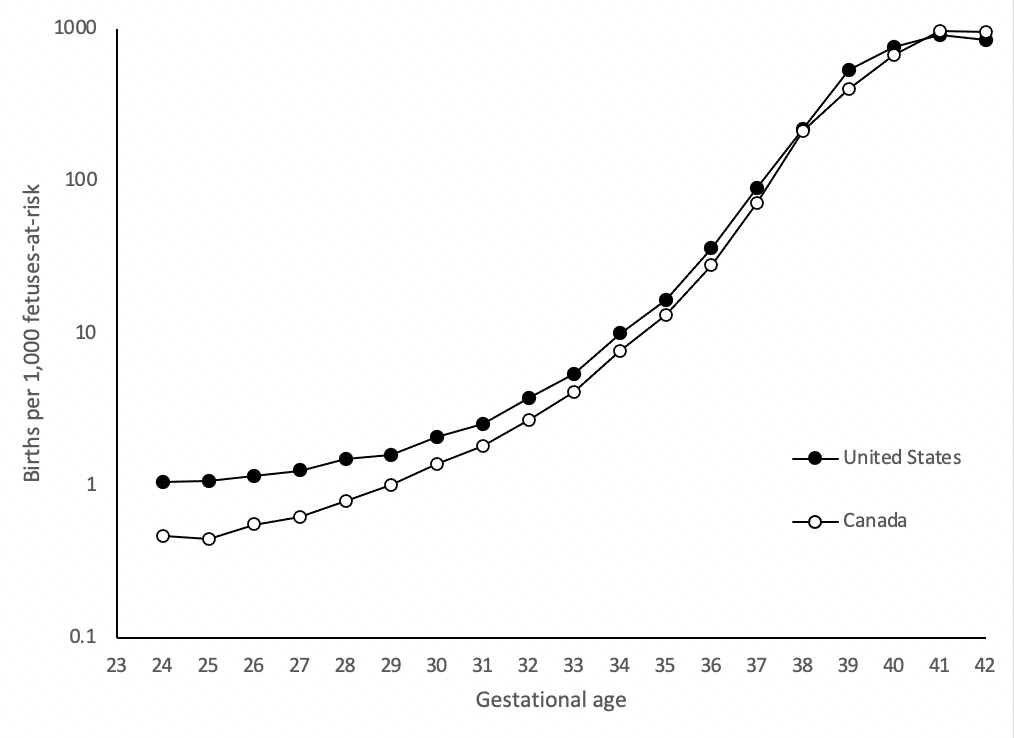

Supplement: S5 Fig — (TIF) [file pone.0253931.s005.tif]

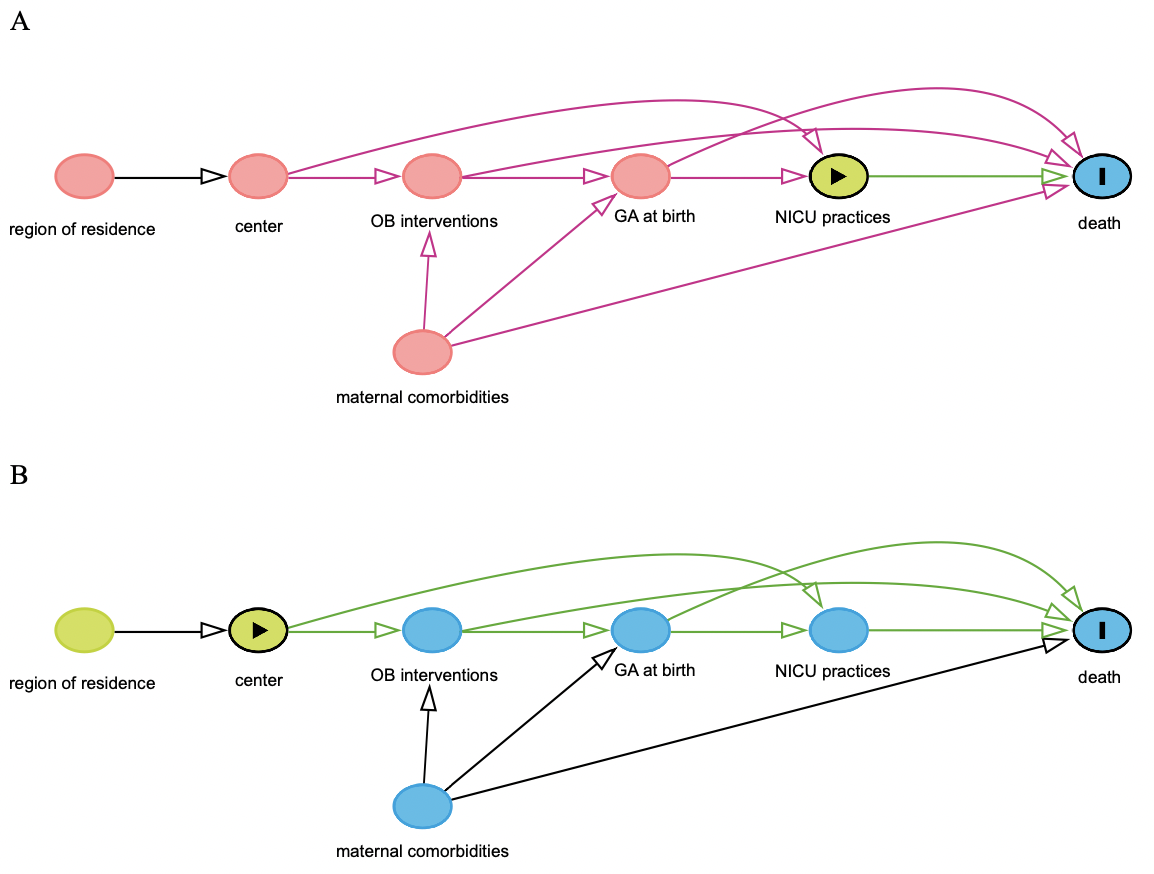

Supplement: S6 Fig — Directed acyclic graph of relationships between NICU practices and perinatal deaths (A) and between hospital or region of residence and perinatal death (B). (TIF) [file pone.0253931.s006.tif]
