# Supplementary material for: Bias in comparisons of mortality among very preterm births: A cohort study
Source: PLoS One. 2021 Jun 30;16(6):e0253931. doi: 10.1371/journal.pone.0253931 (PMC8244917; doi:10.1371/journal.pone.0253931)
Supplement: S1 Table — (PDF) [file pone.0253931.s007.pdf]

S1 Table. Comparisons of rates of early neonatal death rates at 24-31 weeks' gestation and overall among singletons with no congenital or chromosomal anomaly, 2006-2015.

	Rate at **24-31 weeks** (95% CI) Births-based calculation (per 1,000 total births/ births)[a]	RR (95% CI)	Rate at **24-31 weeks** (95% CI) Fetuses-at-risk calculation (per 1,000 fetuses-at-risk)[b]	RR (95% CI)	Rate, **overall** (95% CI) (per 1,000 total births)[c]	RR (95% CI)
Hypertensive disorders in pregnancy [d]						
No	55.9 (55.1 to 56.7)	Ref	0.51 (0.50 to 0.52)	Ref	1.08 (1.07 to 1.09)	Ref
Yes	33.7 (32.5 to 34.9)	0.60 (0.58 to 0.63)	1.24 (1.20 to 1.29)	2.44 (2.35 to 2.54)	1.97 (1.91 to 2.02)	1.83 (1.77 to 1.88)
Maternal race/ethnicity [d]						
White	54.5 (53.6 to 55.3)	Ref	0.48 (0.47 to 0.48)	Ref	1.05 (1.03 to 1.06)	Ref
Black	46.8 (45.7 to 48.0)	0.86 (0.84 to 0.89)	1.01 (0.98 to 1.03)	2.11 (2.05 to 2.18)	1.68 (1.65 to 1.71)	1.61 (1.57 to 1.64)
Native American	52.4 (46.3 to 58.5)	0.96 (0.86 to 1.08)	0.59 (0.52 to 0.66)	1.23 (1.09 to 1.39)	1.31 (1.20 to 1.41)	1.25 (1.15 to 1.35)
Asian	47.9 (45.1 to 50.8)	0.88 (0.83 to 0.94)	0.40 (0.38 to 0.43)	0.84 (0.79 to 0.90)	0.88 (0.84 to 0.92)	0.84 (0.81 to 0.88)
Country of birth						
United States	51.7 (51.0 to 52.4)	Ref	0.56 (0.55 to 0.56)	Ref	1.14 (1.13 to 1.15)	Ref
Canada	54.4 (50.8 to 57.9)	1.05 (0.98 to 1.12)	0.32 (0.30 to 0.34)	0.57 (0.53 to 0.61)	0.60 (0.57 to 0.63)	0.53 (0.50 to 0.55)

[a] Births-based death rates represent proportions, with the number of early neonatal deaths at 24-31 weeks in the numerator and the number of live births at 24-31 weeks in the denominator.
[b] Fetuses-at-risk rates represent cumulative incidence rates with the number of early neonatal deaths at 24-31 weeks in the numerator and the number of fetuses at risk of death at 24 weeks (i.e., fetuses who were delivered at 24 weeks or later) in the denominator.
[c] This calculation is identical whether a births-based/fetuses-at risk calculation is used.
[d] Based on births in the United States.
Legend: *CI* denotes confidence intervals; *RR* denotes rate ratios.
