# Supplementary material for: Bias in comparisons of mortality among very preterm births: A cohort study
Source: PLoS One. 2021 Jun 30;16(6):e0253931. doi: 10.1371/journal.pone.0253931 (PMC8244917; doi:10.1371/journal.pone.0253931)
Supplement: S1 Appendix — (PDF) [file pone.0253931.s008.pdf]

S1 Appendix
Example of an antenatal exposure associated with gestational age at birth, resulting in a paradoxical association with mortality among very preterm births

We illustrated a cohort study of X, an antenatal risk factor. Exposed (yellow) and non-exposed (white) participants are followed until 1 week after birth or death, whichever occurs first. The figure below shows different possible scenarios during the follow-up of fetuses/neonates from a cohort of 20,000 women and fetuses (10,000 exposed and 10,000 non-exposed to X). The number of participants (N) for each possible trajectory is presented in the last column of the table below.

Among fetuses exposed to X (all 6 possible trajectories represented in yellow in the figure), the perinatal mortality rate at 24-31 weeks is 46 per 10,000 fetuses-at-risk, while the rate among non-exposed fetuses (in white) is 17 per 10,000 fetuses-at-risk. A larger proportion of fetuses exposed to X are born before 32 weeks than in the non-exposed group (402/10,000 vs 103/10,000). The lower mortality among very preterm births who are exposed (46/402 or 10.4%) compared with non-exposed (17/103 or 16.5%) does not translate in a survival benefit from exposure to the antenatal risk factor X.

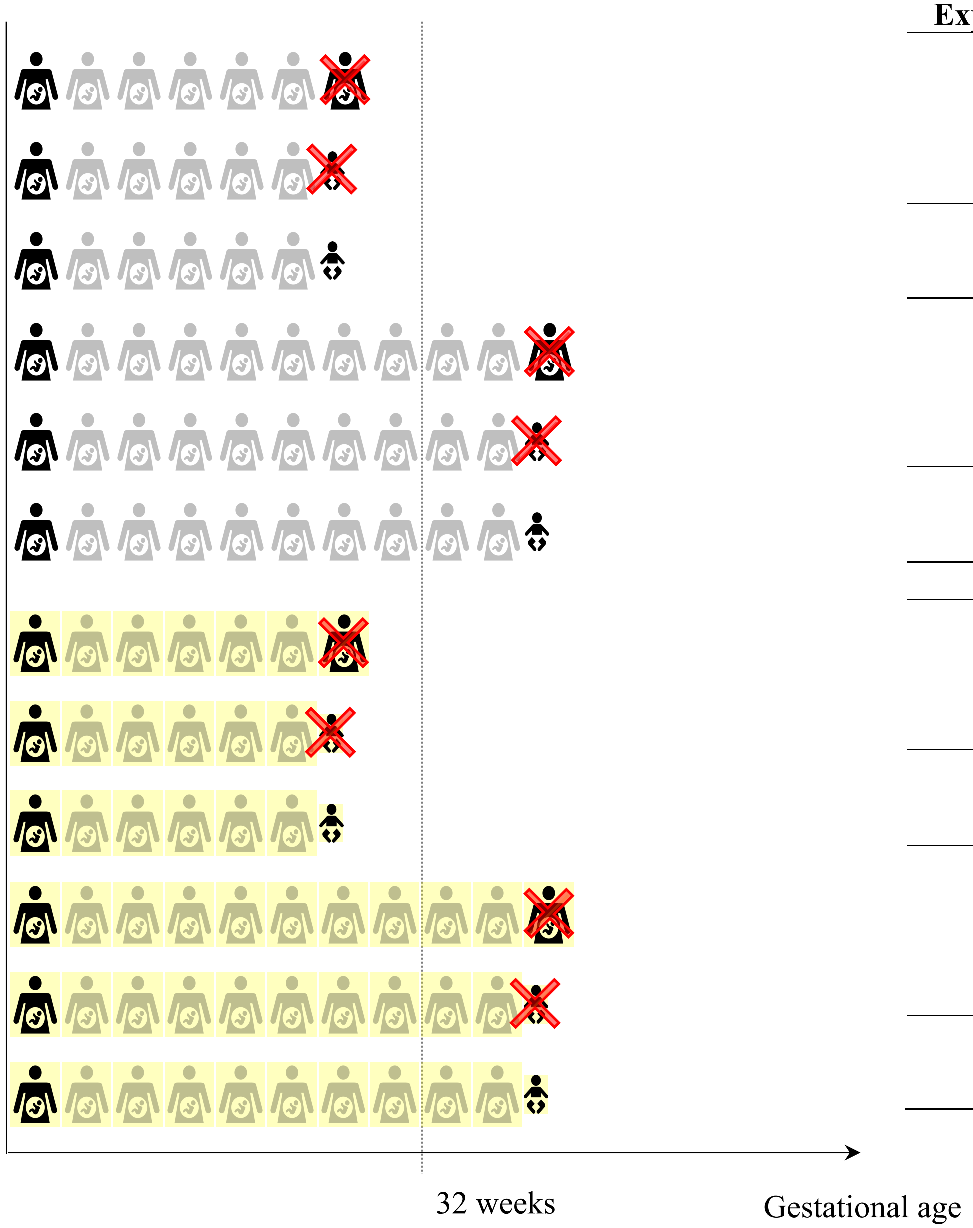

Exposure to X	Gestational age at birth	Outcome	N
No	Birth at 24-31 wks	Early neonatal death or Fetal death	17
No	Birth at 24-31 wks	Survival	86
No	Birth at 32+ wks	Early neonatal death or Fetal death	24
No	Birth at 32+ wks	Survival	9873
Yes	Birth at 24-31 wks	Early neonatal death or Fetal death	46
Yes	Birth at 24-31 wks	Survival	356
Yes	Birth at 32+ wks	Early neonatal death or Fetal death	43
Yes	Birth at 32+ wks	Survival	9555

Numbers in the example above is based on the distribution of births at 24-31 week' gestation and perinatal deaths among mothers of singletons with no congenital or chromosomal anomalies, with or without hypertensive disorders of pregnancy.

	Gestational age at birth	Outcome	N	Proportion of population, by exposure group (‰)	Proportion of population, by exposure group (%)	Mortality per 1,000 births in the same gestational age group	Mortality per 1,000 fetuses-at-risk
No HDP	Birth at 24-31 wks	Perinatal death	65,237	**1.7**	1.0	171	1.8
No HDP	Birth at 24-31 wks	Survival	317,233	**8.6**		-	-
No HDP	Birth at 32+ wks	Perinatal death	87,810	**2.4**	99.0	2	2.4
No HDP	Birth at 32+ wks	Survival	36,420,664	**987.3**		-	-
HDP	Birth at 24-31 wks	Perinatal death	10,326	**4.6**	4.0	113	4.6
HDP	Birth at 24-31 wks	Survival	80,698	**35.6**		-	-
HDP	Birth at 32+ wks	Perinatal death	9,742	**4.3**	96.0	4	4.5
HDP	Birth at 32+ wks	Survival	2,164,551	**955.5**		-	-

The following table reports the distribution of births at 24-31 week' gestation and perinatal deaths among mothers of singletons with no congenital or chromosomal anomalies, in the U.S. and in Canada. A larger proportion of fetuses were born at 24-31 week' gestation in the U.S. than in Canada. Similarly, to the example above, the risk of perinatal mortality at 24-31 week' gestation was higher in the U.S. than in Canada when the whole cohort is considered. Restriction of the cohort to very preterm births lead to a bias when comparing perinatal mortality between the two countries.

	Gestational age at birth	Outcome	N	Proportion of the population (‰)	Proportion of the population (%)	Perinatal deaths per 1,000 births in the same gestational age group	Perinatal deaths per 1,000 fetuses-at-risk
U.S.	Birth at 24-31 wks	Perinatal death	79,570	**2.0**	1.2	166	2.0
U.S.	Birth at 24-31 wks	Survival	400,901	**10.2**		-	-
U.S.	Birth at 32+ wks	Perinatal death	102,061	**2.6**	98.8	3	2.6
U.S.	Birth at 32+ wks	Survival	38,897,820	**985.2**		-	-

CA	Birth at 24-31 wks	Perinatal death	4,363	**1.6**	0.7	226	1.6
CA	Birth at 24-31 wks	Survival	14,939	**5.5**		-	-
CA	Birth at 32+ wks	Perinatal death	7,294	**2.7**	99.3	3	2.7
CA	Birth at 32+ wks	Survival	2,688,573	**990.2**		-	-
