# Supplementary material for: Bias in comparisons of mortality among very preterm births: A cohort study
Source: PLoS One. 2021 Jun 30;16(6):e0253931. doi: 10.1371/journal.pone.0253931 (PMC8244917; doi:10.1371/journal.pone.0253931)
Supplement: Attachment — Submitted filename: PONE-D-20-40633_Response to Reviewers_20210509.docx [file pone.0253931.s009.docx]

**Response to Reviewers**

We thank the Reviewers for their thoughtful review and helpful comments. Our answers to the comments can be found below.

Reviewers' comments:

R1	Reviewer #1: This is a well-written manuscript that examines the differences in how 3 risk factors (maternal hypertension, maternal race, and U.S. vs Canada country of birth) are associated with perinatal and neonatal mortality when analyzed using different subsets of births (limited to births at 24-31 weeks gestation or to all births ≥24 weeks) and with different denominators (among births at the same gestational age week or by a fetuses-at-risk approach). Although it is a methodology-focused manuscript, it is written in a language that should be accessible to both clinicians and clinical researchers. Study data are from population-based datasets based on vital statistics in the U.S. and Canada. The authors show that the methodologic choices described above (subsetting births by a gestational age range and using a denominator based on births or potential births [fetuses-at-risk]) provide paradoxical-seeming results. The findings are important, particularly, as the authors point out, because of the common use of cohort studies based on subsets of births in perinatal/neonatal medicine and epidemiology (example cohorts with hundreds of papers between them that have influenced modern obstetric/neonatology practice: Canadian Neonatal Network, NICHD Neonatal Research Network, iNEO, Vermont Oxford Network, etc). The authors should be commended for a simple demonstration of a complex subject.

A1	Thank you.

MAJOR COMMENTS:

R1.1	The conclusion "Studies of perinatal risk factors and between-centre or between-country comparisons of perinatal mortality lead to biased inferences when restricted to very preterm births" seems overly simplistic. Similarly, there are several instances of this language in the Discussion (e.g., "Our study shows that comparisons of perinatal mortality between fetuses of mothers with and without hypertensive disorders of pregnancy, between mothers of different ethnicity, and between mothers in Canada and the United States, were seriously biased when the study population was restricted to very preterm births and analyses were based on births-based denominators.")

A1.1	Our conclusion applies to a causal context, in which the restriction of a study to very preterm births will lead to biased inferences when evaluating antenatal exposures, or regional differences, that are associated with the birth rate. We now clarify that results of studies restricted to preterm births lead to erroneous conclusions in causal context, but that such restriction could be appropriate in predictive contexts.
We have revised the manuscript as follows:
‘Studies of prenatal risk factors and between-centre or between-country comparisons of perinatal mortality bias causal inferences when restricted to truncated cohorts of very preterm births.’ (line 41)
‘Our study shows that, in research with a cause-and-effect focus, comparisons of perinatal mortality between fetuses of mothers with and without hypertensive disorders of pregnancy, between mothers of different races, and between mothers in Canada and the United States, are seriously biased when the study population is restricted to very preterm births and analyses are based on births-based denominators.’ (line 266)
‘Births-based rates are not inherently problematic as they can be appropriate for prognostication (since they quantify the mortality experience of very preterm infants of women who are older, smoke, or have hypertension, for example). However, such rates are a poor basis for causal inference regarding prenatal factors (such as older maternal age, smoking and hypertension), addressing health inequities and evaluating neonatal health services (through between-centre and between-region comparisons).’ (line 343)

R1.2	The authors might further explore the reasons for the paradoxical associations that they observe. They acknowledge the rich literature on "collider stratification bias" and the theoretical mechanistic model of the "accelerated birth rate" whereby infants in some subgroups who are at lower risk are born earlier and so these subgroup-defining traits appear protective at low gestational ages.

A1.2	In addition to the citation of relevant articles that explain the detailed mechanisms underlying the paradoxical associations we observe, we have added a visual representation of the cohort in an Appendix, along with data from our study. This Appendix illustrates how an accelerated birth rate can inflate the denominator and how an unfavourable exposure can appear beneficial.

R1.3	However, there are alternative explanations for the authors findings that do not fall squarely in the realm of bias. As a clinical neonatologist, I care for infants who are born at early gestational ages. From my point of view, among the very preterm babies admitted to our unit in any given week, birth secondary to maternal hypertensive disease is a positive prognostic sign (compared to the alternative, which is usually birth secondary to some maternal inflammatory/infectious process). Is that point of view (from the neonatologist or perinatologist who delivers or cares for the baby) not valid? The authors allude to the difference between prognostic (my perspective on how the baby will do) and causal models (concerned with etiologic risks), but this might be further explored. From the perspective of the neonatologist/perinatologist, the comparison for births born due to maternal hypertension is not healthy normal pregnancies/births - rather, the comparison is to other births due pathology.

A1.3	We agree with the reviewer that the bias we describe is not inherent to the birth-based calculation, but to the context in which it is used and to the erroneous interpretation of the results. Comparing risk factors among infants born preterm can be appropriate in a predictive context. However, predictions are appropriate for prognostication—not causal inference. Our manuscript highlights the problems related to comparing these frequency measures in a cause-and-effect context. Prediction of better outcome cannot be directly interpreted as a “benefit” of exposure. This distinction is perhaps better illustrated with the finding that very preterm infants of mothers who smoke in pregnancy have lower neonatal mortality rates than very preterm infants of mothers who did not smoke. In this example as well, the predictive finding holds, despite the well-known deleterious effect of maternal smoking on fetuses, irrespective of gestational age at birth. Birth-based formulations in studies restricted to very preterm births can obscure the potential benefit of preventive measures targeting antenatal exposures (e.g., programs to reduce smoking in pregnancy, prevention of hypertensive disorders of pregnancy, etc.) in improving perinatal mortality at 24-31 weeks’ gestation.

We have added:
‘Such restriction results in a right truncation and analysis of incomplete data from the original cohort of individuals exposed or unexposed to a risk factor in pregnancy (see the Appendix for an illustrated example). Births-based rates are not inherently problematic as they can be appropriate for prognostication (since they quantify the mortality experience of very preterm infants of women who are older, smoke, or have hypertension, for example). However, such rates are a poor basis for causal inference regarding prenatal factors (such as older maternal age, smoking and hypertension), addressing health inequities and evaluating neonatal health services (through between-centre and between-region comparisons).’ (line 340)
‘The causation vs prediction dichotomy may be best illustrated by the effect of maternal smoking on neonatal mortality [16]: it is preferable to frame the lower mortality rates among low birth weight and preterm infants of mothers who smoke in pregnancy in prognostic (predictive) terms. It is well-understood that maternal smoking has deleterious effects on the fetus and infants, irrespective of the gestational age at birth.’ (line 348)

As mentioned above, we have added an Appendix in the revised manuscript that should clarify how a better prognosis associated with a risk factor in a subgroup of individuals does not translate into an actual benefit of exposure. This is illustrated with reference to a hypothetical randomized trial for evaluating the effect of a prenatal exposure in pregnancy. In that case, we would contrast the groups of fetuses exposed vs not exposed. Both groups would be followed until a specific time (1 week after birth or death, whichever occurs first). If birth at earlier gestational age is an effect of exposure, we would expect a larger number of preterm births among the exposed group. From a causal point of view, it would be inappropriate to exclude fetuses born at term in the evaluation of the effect of the prenatal exposure on perinatal mortality.

R1.4	Another alternative point of view to consider is that of effect modification. Consider the example of a persistent patent ductus arteriosus (PDA), a common presentation in premature infants that may result in morbidity. In the aggregate, persistent PDA is associated with morbidity. But, in the particular, there are conditions (e.g., transposition of the great arteries) that affect a minority of the group in which the PDA confers a major protective advantage. In a similar way, is it not possible that while, in the aggregate, birth in Canada confers a lower risk of mortality than in the U.S. -- at the same time, care for very preterm births (due to a more aggressive/intensive/ expensive approach) in the U.S. (versus Canada) confers a lower risk of mortality for this minority of patients?

A1.4	We appreciate the reviewers’ comment and suggested explanation. This explanation is predicated on a causal effect. We conceptualize gestational age at birth as a mediator of the causal effect of exposure on outcome.

If we take the example of regional comparisons, we can think of regional factors such as differences in obstetric practices or antenatal care that could influence the birth rate. Our regional comparisons were conducted to infer cause and effect. Regions are a proxy for variations in health care systems, for example. Owing to the universal health care system in Canada, a higher proportion of Canadian women will seek antenatal care early in pregnancy than in the United States. This could, in turn, impact the birth rate, with a higher birth rate in the U.S. than in Canada. Gestational age at delivery/birth was associated with country of residence in our study, and we thus considered gestational age at birth as a mediator of the effects of various exposures related to country of birth on perinatal mortality.

It would be correct to infer that fetuses born early will have better survival in the U.S. than in Canada. However, the likely explanations include a greater risk of earlier births (including early births of less compromised fetuses) in the U.S. than in Canada, and more aggressive obstetrical and neonatal practices, or a mixture of these explanations. The lower perinatal mortality in the U.S. is conditional on being born prematurely, which in turn is associated with a higher risk of perinatal death compared with term birth. If we analyze the complete cohort of pregnant mothers and fetuses reaching 24 week’s gestation, we see that for a 24-week pregnant woman, the risk of experiencing a perinatal loss before 32 weeks of pregnancy is greater in the U.S. than in Canada.

We have added the following text to the revised manuscript in an effort to address these issues:
‘Although studies may be restricted to very preterm infants with the intent of comparing NICU practices (S6a Fig), the contrast of delivery hospital or regional rankings with regard to fetal and neonatal mortality at early gestational age can be similarly biased by differential distributions of gestational age at birth (S6b Fig), which is an intermediate factor between region or hospital admission and perinatal death Distributions of gestational age at birth could differ between regions due to variations in obstetrical and neonatal practices and access to care for example.’ (line 328)

We have also added another explanation for the paradox discussed in the literature that involves a cortisol-mediated mechanism.
‘The problem of the paradox of perinatal mortality curves has also been framed as a collider stratification bias [19] (i.e., restricting by birth weight or gestational age, which are common effects of the perinatal determinants and unmeasured confounders, introduces a bias), and as effect-modification due to cortisol-mediated intrauterine adaptation resulting from the chronic stress associated with preterm birth.[38] However, the paradox of intersecting perinatal mortality curves manifests across a range of contrasts (including maternal age, smoking, hypertension, race, and country), and although it is possible to postulate a mechanism for the reduced mortality at preterm gestation associated with one factor or other, the need to propose a mechanism for each of these diverse contrasts supports the more parsimonious, singular explanation that the birth-based analysis is flawed.’ (line 291)

R1.5	The authors should explore reasons for their findings that are not simply the result of bias. Bias implies that these other perspectives are erroneous, which is not clearly the case. They might consider a more nuanced elaboration of how to interpret their findings in light of the research/clinical question being asked.

A1.5	The research question is definitely crucial in choosing the right type of analysis and interpreting the results properly. The problem we identify and the examples we used are all within the context of explanatory analyses and causal interpretations.
‘In this study, we analyze the effects of prenatal exposures on perinatal outcomes.’ (line 80)

In the revised manuscript, we acknowledge that the paradoxical effects are valid from a prediction/prognostic standpoint.
‘Births-based rates are not inherently problematic as they can be appropriate for prognostication (since they quantify the mortality experience of very preterm infants of women who are older, smoke, or have hypertension, for example). However, such rates are a poor basis for causal inference regarding prenatal factors (such as older maternal age, smoking and hypertension), addressing health inequities and evaluating neonatal health services (through between-centre and between-region comparisons).’ (line 343)

We also acknowledge alternative explanations in the literature which refer to these phenomena.
‘The problem of the paradox of perinatal mortality curves has also been framed as a collider stratification bias[19] (i.e., restricting by birth weight or gestational age, which are common effects of the perinatal determinants and unmeasured confounders, introduces a bias), and as effect-modification due to cortisol-mediated intrauterine adaptation resulting from the chronic stress associated with preterm birth.[38]’ (line 291).

MINOR COMMENTS

R1.6	Ln 33 - specify that this is the perinatal (or neonatal) death rate

A1.6	We specify the reported ratios refer to comparisons of perinatal death rates:
‘However, overall (≥24 weeks) perinatal death rates were higher among births to hypertensive vs non-hypertensive women (RR 2.14, 95% CI 2.10-2.17), Black vs White mothers (RR 1.86, 95% CI 1.84-1.88;) and births in the United States vs Canada (RR 1.08, 95% CI 1.05-1.10), as were perinatal death rates based on fetuses-at-risk at 24-31 weeks (RR for hypertensive disorders: 2.58, 95% CI 2.53-2.63; RR for Black vs White ethnicity: 2.29, 95% CI 2.25-2.32; RR for United States vs Canada: 1.27, 95% CI 1.22-1.30).’ (line 35)

R1.7	Ln 86 - consider removing phrase “very poor survival.” In some cohorts, survival exceeds >50% at 22 and 23 weeks. The other reasons are valid enough without this one.

A1.7	We have removed this phrase.

R1.8	Table 1 - some of the cell totals for no hypertensive disorders in pregnancy are greater than the overall totals for those rows

A1.8	We have made the appropriate corrections and have verified all results to ensure no other similar errors occurred when copying results from our Excel files to the manuscript tables.

R1.9	Table 2 - I found the inclusion of "Births- based/fetuses- at risk calculation (per 1,000 total births)" under "overall" perinatal death rate confusing. (Same applies to S1 Table.) I think that the authors are trying to demonstrate that these formulations are the same. But, because this is already specified in the text, perhaps they should simply write "Calculation per 1,000 total births" (easier to read/less confusing) and, if needed, use a footnote to remind the reader that "for the overall calculation, births-based and fetuses-at-risk formulations are equivalent."

A1.9	The phrase ‘Births-based/fetuses-at risk calculation’ has been moved to a footnote, as suggested.

R1.10	Ln 283 - why write "though not likely"? Is that quantifiable (as in an E-value?) or is it an opinion?

A1.10	Other confounders, not included in this analysis, would be expected to confound the maternal hypertension-perinatal death relationship to a lesser extent than maternal age and diabetes which had limited impact on the rate ratios. We have calculated E-values (which ranged from 2.6 to 4.6) and have added an explanation for our comment in the Discussion:
‘It is possible, though not likely, that other factors such as parity, chronic diseases and socioeconomic status could have confounded the association to a greater extent since adjustment for maternal age and diabetes, two well-known confounders of the relationship between hypertensive disorders and outcomes, resulted in a 5 to 11% change in the rate ratios. Strong unmeasured confounders would be required to nullify or change the direction of the association.’ (line 381)

R1.11	Ln 293 - as noted above, perhaps "potential bias"? Or some more nuanced interpretation?

A1.11	Although it may not always result in an inverse association, restricting a study sample to very preterm live births does not allow the estimation of the total effect of a prenatal exposure on subsequent outcome, owing to the exclusion of stillbirths, earlier preterm births, and the right truncation (exclusion of later-born fetuses) of the cohort.

We have clarified our conclusion as follows:
‘Our analyses highlight the bias inherent in studies restricted to very preterm birth subpopulations that aim to estimate the causal effect of prenatal risk factors, social determinants of health and between-country and between-centre comparisons.’ (line 394)

R1.12	S3Fig - should the y-axis be for "fetuses-at-risk" (same as S4 and S5Fig)?

A1.12	We have modified the axis title as suggested.

R2	Reviewer #2: This paper is extremely well written and tackles a very important issue around bias in perinatal mortality and neonatal mortality rates. It takes the research around analysis of births versus fetuses at risk in an important direction and is written in an easy to understand style with clear diagrams.

A2	Thank you.

R2.1	I have only a few minor points:
1. A definition of early neonatal death and perinatal death used here would be extremely helpful as there some slight variation in the use of these terms.

A2.1	We now specify:
‘Dependant variable
The primary outcome was perinatal death, defined as stillbirth or early neonatal death (i.e., death within 7 days of delivery). The secondary outcome was early neonatal death.’ (line 116)

R2.2	Some of the graphs may benefit from alternative plotting shapes to add differentiation of the lines as the colours are hard to differentiate e.g. triangles and squares to differentiate between the ethnic group data that is very closely aligned

A2.2	We have modified the figures as recommended by the reviewer.

R2.3	More detailed description of why the biases might arise would benefit the discussion and make it easier to interpret with perhaps examples.

A2.3	We have added an Appendix to illustrate the bias.
